# A Few “Buts”

**Published:** 1908-06

**Authors:** 


### Abstracts and Selections.

### A Few "Buts."

"Just keep your heart a beating warm,
Be kind to every fellow;
Look for rainbows in the storm,—
But carry an umbrella."

"Be strong to battle in the strife,
Be true when others doubt you;
Don't think that money's all in life,—
But carry some about you."

"And when 'tis time to shuffle off,
And you have done your mission,
Just put your trust in Providence,—
But call a good physician."

"And when you settle up accounts,
Prepared to shun life's ills,
Please do not act as many do,—
But pay your doctor bills."

[The "Red Back" is indebted to Dr. Wilkins, of Wellington, Texas, for the above.]

